# Evaluation of the Effectiveness and Safety of the BNT162b2 COVID-19 Vaccine in the Vaccination Campaign among the Health Workers of Fondazione Policlinico Universitario Agostino Gemelli IRCCS

**DOI:** 10.3390/ijerph182111098

**Published:** 2021-10-22

**Authors:** Domenico Pascucci, Mario Cesare Nurchis, Martina Sapienza, Francesco Castrini, Flavia Beccia, Floriana D’Ambrosio, Adriano Grossi, Carolina Castagna, Angelo Maria Pezzullo, Maurizio Zega, Domenico Staiti, Francesco Maria De Simone, Nadia Mores, Andrea Cambieri, Giuseppe Vetrugno, Gianfranco Damiani, Patrizia Laurenti

**Affiliations:** 1 Università Cattolica del Sacro Cuore, 00168 Rome, Italy; domenico.pascucci@outlook.it (D.P.); martina.sapienza93@gmail.com (M.S.); fr.castrini@gmail.com (F.C.); flavia.beccia@gmail.com (F.B.); florianadambrosio@libero.it (F.D.); adriano.grossi@yahoo.it (A.G.); carolina.castagna@gmail.com (C.C.); angelomaria.pezzullo@unicatt.it (A.M.P.); domenico.staiti@unicatt.it (D.S.); nadia.mores@policlinicogemelli.it (N.M.); giuseppe.vetrugno@policlinicogemelli.it (G.V.); Gianfranco.Damiani@unicatt.it (G.D.); patrizia.laurenti@policlinicogemelli.it (P.L.); 2 Fondazione Policlinico Universitario A. Gemelli IRCCS, 00168 Rome, Italy; maurizio.zega@policlinicogemelli.it (M.Z.); francescomaria.desimone@policlinicogemelli.it (F.M.D.S.); andrea.cambieri@policlinicogemelli.it (A.C.)

**Keywords:** effectiveness, safety, health workers, vaccine, vaccination campaign, COVID-19

## Abstract

Health workers, especially those in patient-facing roles, had a significantly increased risk of COVID-19 infection, having serious outcomes, and risking spreading the virus to patients and staff. Vaccination campaign planning suggests allocating initial supplies of BNT162b2 vaccine to health workers given the importance of early protection to safeguard the continuity of care to patients. The aim of the study is to assess the effectiveness and safety of BNT162b2 vaccine among the health workers of Fondazione Policlinico Universitario Agostino Gemelli IRCCS (FPG). The retrospective cohort study was conducted among health staff working at the FPG. Vaccination data were collected from hospital records. The primary end points were vaccine effectiveness and safety. A total of 6649 health workers were included, of whom 5162 received injections. There were 14 cases of COVID-19 with onset at least 14 days after the second dose among vaccinated health workers and 45 cases among unvaccinated ones. BNT162b2 was 91.5% effective against COVID-19 (95% credible interval, 84.7% to 95.3%). The safety profile of BNT162b2 vaccine consisted of short-term, non-serious events. The promotion and boost of the COVID-19 vaccination campaign represents a key public health measure useful to curb the spread of the pandemic especially in vulnerable contexts, such as hospitals, where health workers carry out a paramount role for the entire community, and requires further protection with a possible booster dose in view of autumn-winter 2021.

## 1. Introduction

On 11 March 2020, the rapidly spreading novel coronavirus outbreak was declared a pandemic by the World Health Organization (WHO). From that moment on, SARS-CoV-2 has affected hundreds of millions of people globally. As of 24 September 2021, as reported by the WHO, there have been 230,418,451 confirmed cases of COVID-19, including 4,724,876 deaths, worldwide [1]. To date, the scientists reported that COVID-19 pandemic had a substantial burden of disease worldwide [2,3,4,5,6].

As an action to curb the burden of COVID-19, Italy authorized the marketing of Pfizer-BioNTech (BNT162b2) vaccine and licensed it for use [7] after it showed high effectiveness and safety against symptomatic infection in a large multinational clinical trial [8]. BNT162b2 is a lipid nanoparticle-formulated, nucleoside-modified RNA vaccine encoding a prefusion-stabilized, membrane-anchored SARS-CoV-2 full-length spike protein [9]. In December 2020, Italy, jointly with other European countries, started the COVID-19 mass vaccination campaign, initially addressed to health workers, staff, and patients of homes for elderly people. Health workers, especially those in patient-facing roles, had a significantly increased risk of COVID-19 infection, having serious outcomes, and risking spreading the virus to patients and staff [10]. Therefore, health workers have been prioritized to receive COVID-19 vaccines in many countries. By the end of March 2021, in the majority of the Italian hospitals, most health workers received both doses of the BNT162b2 COVID-19 vaccine, while few received both doses of mRNA-1273 (i.e., Moderna). In Italy, as of 25 September 2021, a total of 83,662,989 vaccine doses were administered [11], vaccinating 77.4% of the population aged over 12.

Historically, vaccination campaigns and, in particular, the one against the ongoing pandemic represent the most efficient cornerstone in achieving potential public health goals [12]. Reduction of pressure on the healthcare system, reduction of overall COVID-19 severity and mortality, re-opening of society, and disease eradication are four main potential targets related to COVID-19 vaccination campaign. Notwithstanding, the strategy behind vaccination campaigns requires efficient and effective planning, coordination, and cooperation between the national and regional health bodies, allowing a unified and centralized response to the country’s immunization plan.

As highlighted by the WHO [13], COVID-19 vaccines have been proven as safe, effective, and life-saving against this severe disease in several large vaccine trials [8,14,15,16]. Several studies have been conducted to assess the effectiveness of vaccination campaigns in the health care workers in real-world settings [17,18,19,20]. We add to this evidence by providing an assessment of the effectiveness and safety of (BNT162b2) vaccine among the health workers of Fondazione Policlinico Universitario Agostino Gemelli IRCCS (FPG), a large teaching hospital in Rome (Italy).

## 2. Materials and Methods

### 2.1. Study Design and Participants

A retrospective cohort study among health staff working at the FPG was conducted to evaluate the effectiveness and safety of BNT162b2 vaccine during the first phase of the vaccination campaign against SARS-CoV-2. All healthcare workers were offered vaccination by the hospital FPG.

Healthcare workers (aged ≥18 years) working at hospital sites who could provide written informed consent and who completed the immunization program with the administration of the second dose after approximately 21 days from the first dose at the FPG were included.

However, not all participants were given two doses; those who had the disease between three months after the date of the first positive swab and within six months of the disease and received only one dose of the vaccine were anyway eligible to join the study. Participants were excluded from this analysis if they either had a positive PCR test after 31 December 2020 and had clinical contraindications to the administration of the vaccine.

Participants were assigned into either the cohort of vaccinated or the cohort of unvaccinated health workers at the beginning of the follow-up period (i.e., 28 December 2020).

The study protocol was approved by the FPG Ethics Committee on 12 July 2021 with the approval number 3973.

### 2.2. Data Sources

Vaccination data were extracted from hospital records for the period of interest, i.e., from 28 December 2020 to 31 March 2021. The time horizon was defined following the Italian ministerial decree no. 44 [21] of April 2021 imposing the compulsory COVID-19 vaccination for all the health workers.

A database was created with an identification code for each participant deriving from the tax code that was unique but anonymous with the indication of the professional category. Sociodemographic data (i.e., age and sex) was retrieved from the employee tax code. Staff occupation information (i.e., physicians, nurses, and other health workers) was obtained from the hospital human resources department.

The date of the first vaccination, the date of the second dose, batch numbers of the two doses, and the reason for the drop out of the second dose have been cataloged.

The data concerning positive swab results (i.e., validation date and type of swab) were extracted through the hospital information systems. Information for adverse drug reactions has been retrieved from the national pharmacovigilance network for FPG.

### 2.3. Effectiveness

Effectiveness of BNT162b2 vaccine against the transmission of SARS-CoV-2 was measured. In particular, a vaccine breakthrough infection is defined as the detection of SARS-CoV-2 RNA or antigen, confirmed by a subsequent PCR test in a respiratory specimen collected from a person ≥14 days after they have completed all recommended doses of a European Medicines Agency-authorized COVID-19 vaccine [22].

FPG offer SARS-CoV-2 testing to all symptomatic and asymptomatic working staff. SARS-CoV-2 PCR testing of combined nasal and oropharyngeal swab specimens was offered to symptomatic staff. Asymptomatic health care workers were invited to participate in voluntary nasal and oropharyngeal swab PCR or antigenic testing. Frequency of voluntary testing was determined according to the occupational risk, on the appearance of COVID-19 like symptoms or being in close-contact with a COVID-19-positive case.

Moreover, unvaccinated health workers who had a positive PCR or antigenic test after less than 14 days from the completion of the immunization program by the first pool of vaccinated individuals (19 January 2021), those who contracted the SARS-CoV-2 infection as ascertained by a positive PCR or antigenic test after the first dose of vaccine, and individuals who suffered from adverse reactions after the first dose and to whom the second dose was not administered precautionary, were excluded from the effectiveness assessment.

### 2.4. Safety

The analysis of the vaccine safety among the health workers vaccinated during the vaccination campaign at FPG was performed on the suspected adverse drug reactions spontaneously reported by the vaccination hub’s team or HCWs retrieved from the national pharmacovigilance network (RNF). For the analysis, we considered the following: number of cases, demographic characteristic of the patients, seriousness, and symptoms reported. The terms for adverse reactions were coded as Preferred Terms (PT), according to the Medical Dictionary for Regulatory Activities (MedDRa), version 24.0.

The sample was composed of all the health workers vaccinated between 28 December 2020 and 21 April 2021, independently from the dose number.

Suspected adverse drug reactions have been spontaneously reported through either the online platform Vigifarmaco or the dedicated AIFA ADR reporting form and sent to the national pharmacovigilance network by the local responsible person for pharmacovigilance platform Vigifarmaco [23]. The information collected by ADR reported after the first dose have been considered to assess the appropriateness for the administration of the second dose by a collaborative team coordinated by the FPG risk management unit, proposing a dedicated path of counselling and allergological/emergency surveillance for second dose administration if the second dose was not contraindicated.

The reported symptoms were compared for frequency to the information available in the Summary of Product Characteristics (SPC) for Comirnaty vaccine (i.e., 24 September 2021), where the adverse reactions are categorized by SOC System Organ Classification and PT according to frequency of occurrence as following in very common (i.e., >10%), common (i.e., between 1% and 10%), not common (i.e., between 0.1% and 1%), rare (i.e., <0.1%), and not known.

### 2.5. Statistical Analysis

A preliminary descriptive analysis focused on frequencies, percentages, and measures of central tendency was performed adopting graphs and tables.

For the first outcome of the analyses, vaccine effectiveness was estimated according to the following formula [24]:

100×(1−IRR)

where IRR is the incidence rate ratio of developing the disease for vaccinated individuals compared with unvaccinated people.

The IRR is given by the formula

$$\mathrm{IRR}=\frac{\mathrm{Vaccinated}\;\mathrm{incidence}\;\mathrm{proportion}}{\mathrm{Unvaccinated}\;\mathrm{incidence}\;\mathrm{proportion}}$$

Vaccine effectiveness is interpreted as the proportionate reduction in disease attack rate among the vaccinated group [25].

The 95% credible interval for vaccine effectiveness was calculated adopting a Bayesian beta-binomial model [8]. According to Bayesian statistics, 95% credible interval was defined as the interval that includes 95% of posterior distribution and is bounded by the 2.5th and 97.5th percentiles [8,26].

In addition, the absolute risk reduction (ARR) was computed as the difference of risks between the vaccinated and unvaccinated groups, while the relative risk reduction (RRR) was estimated as the ratio of the difference of risks between the vaccinated and unvaccinated groups to the risk of events in the unvaccinated group.

Regarding the safety analysis, the results of vaccine safety were descriptive in nature. Categorical data are presented as absolute and relative number of patients. For continuous data, mean and standard deviation (SD) or median with interquartile range (i.e., 1st quartile and 3rd quartile) was used, depending on its distribution.

In addition, a multivariate logistic regression was run to investigate the factors, such as gender, age, and occupation, influencing the likelihood towards vaccination. According to the assumption of the chosen model, the variable identifying the likelihood towards vaccination was dichotomized into health workers who got vaccinated and those who did not.

All statistical analyses were conducted with a statistical significance level set at *p* < 0.05 and performed with STATA 16 (StataCorp LP, College Station, TX, USA) and Python programming language (version 3.9.7, Python Software Foundation, http://www.python.org (accessed on 10 September 2021).

## 3. Results

### 3.1. Participants

Between 28 December 2020 and 31 March 2021, a total of 6649 health workers were observed.

For effectiveness assessment, among the vaccinated health workers, 79 were excluded from the analysis, of which 44 were excluded due to SARS-CoV-2 infection after the first dose of vaccine, 25 due to adverse reactions to the first dose and to whom the second dose was not administered, and 10 due to SARS-CoV-2 infection within 14 days after the second dose of vaccine. Thus, 6570 health workers were included in the analysis. A total of 5152 were vaccinated, while 1418 were unvaccinated. Among these participants, 61% were female, 41% were older than 40 years of age, and 45% were physicians. The median age at vaccination was 39 years (Table 1).

For safety evaluation, 152 Individual Case Safety Reports (ICSRs) were collected from the sample of 5231 vaccinated people. The median age was 42 (IQR 30–50). Among the respondents, 81% were females.

Among health workers who have completed the immunization program, the vaccine coverage was 78% on 31 March 2021. A multivariate logistic regression was run to investigate the factors associated with the uptake of health workers towards vaccination. The findings depicted that being aged between 40 and 59, compared with the youngest age band, was statistically significant (OR 1.17, 95% CI 1.04–1.34) associated with the uptake to vaccination as well as being a physician (OR 2.23, 95% CI 1.92–2.59) and nurse (OR 1.88, 95% CI 1.61–2.19) in comparison with being another health worker. The gender is not significantly (OR 1.05, 95% CI 0.92–1.19) associated with the uptake to vaccination.

### 3.2. Effectiveness

Among 6570 health workers without evidence of existing or prior SARS-CoV-2 infection, 14 cases of COVID-19 with onset at least 14 days after the second dose were observed among the vaccinated individuals and 45 among unvaccinated ones. This case distribution corresponds to 91.5% vaccine effectiveness (95% CI, 84.7% to 95.3%).

The absolute risk reduction and the relative risk reduction amounted to 2.9% and 91.4%, respectively.

### 3.3. Safety

Twelve percent of subjects reported only one symptom, whereas 88% reported more than one. Eighty five percent of ICSRs were reported from healthcare professionals under 55 years of age.

Thirty-seven per cent of patients had comorbidity. Sixty-eight per cent of patients reported adverse reactions in the same day of the vaccination, whereas 24% within 48 h. Only one ADR report was compiled at the tenth day after vaccination.

A major part of ADRs consisted of non-serious events. Only six of them (4%) were reported as serious. These cases were marked by angioedema, odynophagia and facial paraesthesia, dysphagia, oedema, urticaria, dyspnea, and respiratory failure. Table 2 depicts the comparison between the data from SPC Comirnaty and vaccinated health workers in FPG.

Among the most common reactions, fever was reported by 39% of respondents, whereas, in the SPC [27], it appears to be less frequent, near 10%. Fever was often accompanied by chills (37%) and fatigue (3%). Headache occurred in 39% of respondents, whereas it was expected to occur in more than 50% of cases. Diarrhea occurred to 5% of subjects. This symptom was not observed during the authorization trials but during post-authorization surveillance. The exact percentage of reference is not known, although it is enlisted among the very common adverse reactions. Arthralgia and myalgia were referred to by 28% of individuals, slightly different from the SPC. Tachycardia was found in 8% of cases. Injection site pain, which was expected to be the most reported adverse reaction, was instead found in 26% of cases. In addition, injection site swelling was found to be uncommon (1%).

As for the common reactions, our findings highlighted higher rates of occurrence for nausea, which was reported by 13% of respondents. Vertigo and injection site redness were found in 5% and 3% of cases, respectively.

With regard to uncommon reactions, lymphadenopathy was present in 15% of cases, insomnia in 2%, malaise in 6%, and injection site pruritus in 3%. Hypersensitivity reactions accounted for 10% of reported symptoms. Pain in extremity, referring to the arm site of injection, occurred in 1% of cases. Acute peripheral facial paralysis, accounted as a rare reaction, was not observed.

Similarly, there were no cases of anaphylaxis. Only one case of myocarditis was reported, as for the extensive swelling of the vaccinated limb. Instead, facial swelling was present in 5% of cases, mostly with perioral oedema and odynophagia.

The ICSRs collected have demonstrated a consistent occurrence of paraesthesia (24%), vertigo (5%), and abdominal pain (3%). Other symptoms, such as localized pain in a specific part of the body, cough, increased blood pressure, drowsiness, and confusion, have been reported sporadically. In addition, the unexpected symptoms (i.e., dyspnea and paraesthesia) observed in our sample were not taken into account by the periodic safety update reports published by the European Medicines Agency [28].

## 4. Discussion

In this large, monocentric retrospective cohort study of hospital workers in Italy, we found high short-term effectiveness of BNT162b2 vaccine against COVID-19 infection. We confirm substantial safety of the BNT162b2 vaccine in a real-world setting with an enhanced pharmacovigilance system, with a higher rate of rare and very rare adverse reactions. Three months after vaccine roll-out started, 78% of health workers received at least one dose of vaccine. The administration of COVID-19 vaccination in FPG was found to be significantly associated with the age class 40–59 and with being a physician or nurse.

As it concerns the effectiveness assessment, our findings (91.5%; 95% CI, 84.7% to 95.3%) were in line with those observed in the scientific literature [29,30]. Moreover, focusing on the hospital setting and on a specific population (i.e., health workers), evidence in the literature showed a slightly lower vaccine effectiveness with respect to our results [17,18,19,20]. The difference in vaccine effectiveness could be explained by the differences in the definition of vaccine breakthrough [22] and also probably by the strict hospital policy and procedures through which it incentivizes the continuous usage of individual protection devices, such as surgical and FFP2 face masks, and the proper physical distancing. In relation to this, the FPG provides FFP2 masks to its health workers daily in order to facilitate the personal protection.

The analysis of the spontaneously reported adverse drug reactions for FPG during the period of interest were consistent for signs and symptoms with those of the two main trials by Polack et al. and the information described at point 4.8 of the vaccine reference document as per the most recently updated SmPC from September 2021, and the COVID-19 safety update for Comirnaty periodically released by EMA. The analysis of reported adverse reactions delineated a safety profile characterized by short-term, mild-to-moderate ADRs, spontaneously regressing within days, confirming the findings emerged from clinical trials. In our study, the local ADR reporting rate was 2.905 ICRs for 100,000 doses, consistently higher than the one described in the fourth COVID-19 vaccine surveillance report of AIFA for the considered time horizon, reporting a rate of 309 ICSRs for 100,000 doses administered, with the FPG reporting rate being almost 10 times higher. This disproportion could be explained by a higher reporting attention and propensity mainly facilitated by the organization of vaccination hub and vaccination team.

A higher reporting rate, in respect to the frequency described in SmPC, was recorded in the study population for hypersensitivity reactions and lymphadenopathy (10% vs. <1% and 15% vs. <1%), although these are expected and usually considered as common symptoms also for vaccines. On the other hand, tachycardia as well as diarrhea were found to be less frequent than expected (8% vs. >60%), respectively. These differences, observed in the frequency of undesired effects, would seem to lean toward a less favorable safety profile than that firstly envisioned by Polack et al., but it should be noted that our population included comorbid vaccines, immune-compromised, or previously COVID-19 infected individuals. Comorbid individuals were underrepresented in the trial by Polack et al. According to our findings, 56 ICSRs (37%) were reported by comorbid patients.

Based on the study results and consistently with the evidence in the scientific literature as well as the indications of regulatory agencies, the BNT162b2 vaccine is to be considered effective and safe. Additionally, the present findings raise an intriguing implication, providing support for a conceptual premise that could be cost-effective [31] for investing resources into the development and adoption of mRNA vaccine platforms given their safety and effectiveness, paving the way to a new era in vaccinology.

Furthermore, the study results have remarkable implications for policymakers in steering choices related to the promotion of the current vaccination campaign among the still unvaccinated health workers but also in the general population by enhancing evidence-based communication strategies.

The findings suggest also that highlighting a point of reference for the vaccine recipient and providing information and monitoring over time the suspected adverse events following immunization allows a deeper awareness and shared knowledge of post-vaccination adverse reactions and consequently support the construction of dedicated and personalized paths improving the personalization and efficiency of care. Comirnaty vaccine is the most widely used and the one for which proportionally there have been more spontaneous reports for adverse events following immunization, the analysis of which have resulted in updating of the SPC for both in frequency and type of undesired effects, confirming the importance and crucial role of post-marketing monitoring of medical products. Merging all available evidence from clinical trials, official repositories, and findings that emerged in a real-world context may in fact provide significant insights on unsolicited adverse events for their prompt management in clinical practice and prevention.

The joint reading of our safety results allows for another conceptual main implication. The results of the study supports the importance of the post-authorization pharmacovigilance activity in general but also specifically for the COVID-19 vaccines, for which the regulatory agencies have set up close post-authorization surveillance systems given the exceptional timeframe observed for the review and authorization processes. The performed collaborative activities at FPG as described in our study could serve as a proposal for policy makers, especially in anticipation of the eventual administration of a third dose for the Comirnaty vaccine.

The findings of the present study must be considered in light of its weaknesses and strengths. In relation to vaccine effectiveness assessment, one important limitation is that our results are not generalizable to the entire population since health workers have a higher risk of infection. However, the considered sample of health workers is quite large, allowing us to perform the statistical procedures. At the beginning of the follow-up period, many of the health workers in the cohort of vaccinated individuals were not vaccinated yet. Thus, the follow-up period is not homogeneous among the participants. Notwithstanding, given that many individuals were vaccinated within the end of January, the follow-up period was approximately two months. A further limit was the lack of stratification by individuals pre-existing medical conditions. Nevertheless, the relatively young age of individuals in the sample suggests that the risk of developing a severe disease after the infection is very low [32,33]. Another limitation lies in the absence of stratification by hospital wards. Indeed, some wards were dedicated only to the management of COVID-19 cases, exposing health workers working in these wards to a higher risk compared with their colleagues.

The strength of this study is given by the systematic and periodic collection of PCR data and clinical symptoms on a cohort of health workers.

As it concerns safety evaluation, the stimulated collection of adverse events exposes the results to the risk of over-reporting. Moreover, the considerations about safety are expressed on a small sample. Anyhow, the methodology implemented at our sample represented, in our opinion, a valuable aid, and the crucial support to the post-marketing pharmacovigilance activity was provided by the activity of the vaccination team at the vaccination hub not only filing and collecting ADR reports but also facilitating and supporting the compilation of the online/paper forms and performing follow-ups of the cases not completely resolved after 48 h or deserving further investigation in the later days after vaccination. In addition, for cases consistent to allergic reactions symptoms or hypersensitivity, a collaborative medical assessment process coordinated by the risk management was carried out with the involvement of the allergology and emergency units to provide vaccines within a comfortable and safe environment.

Pharmacovigilance descriptive analysis for adverse event following immunization presented in this study may suffer of the commonly cited limitations, including, among others, no definitive proof of causal relationship between exposure to the medical product and the reported event occurrence, the potential bias due to underreporting, passive reporting, stimulated reporting, and other confounding factors. Furthermore, the lack of a denominator for the analysis of spontaneous reporting system databases does not allow absolute measures of risk estimation [34,35]. Nevertheless, the results presented in this study suggested differences in frequency of expected events since the adverse reactions are always characterized by mild-to-moderate symptoms and mostly completely resolved within a week after vaccination. Furthermore, due to the activation of a clinical dedicated path for subjects reporting allergic symptoms, prevention for related adverse events could be expected, and in fact, none of the subjects reported severe adverse reactions.

At the time of our study, the most frequent lineage in Italy was the B.1.1.7 (i.e., alpha); thus, further studies are needed to evaluate the vaccine effectiveness against the new rising variants of concern and the related period of protection from the infection. Moreover, additional research is needed to investigate and validate a cut off according to which individuals have a higher risk of being infected with respect to others.

## 5. Conclusions

In the present pandemic context, the BNT162b2 vaccination, jointly with other public health measures [36], allows to lessen the impact of COVID-19 on health and economic and social well-being. Thus, in a perspective of sustainability of the National Health Service, it is essential to promote the current vaccination campaign in order to curb the spread of COVID-19 especially in vulnerable contexts, such as hospitals, where health workers play a paramount role for the entire community and require further protection with a possible booster dose in view of autumn and winter 2021.

## Figures and Tables

**Table 1:** Demographic characteristics of health workers.

Characteristics	Vaccinated (N = 5152)	Unvaccinated (N = 1418)	Total (N = 6570)
**Sex—no. (%)**			
Male	1999 (39)	533 (38)	2532 (39)
Female	3153 (61)	885 (62)	4038 (61)
**Age—no. (%)**			
20–39	2582 (50)	725 (51)	3307 (50)
40–59	2136 (41)	590 (42)	2726 (41)
60–76	434 (8)	103 (7)	537 (8)
**Occupation—no. (%)**			
Physicians	2415 (47)	521 (37)	2936 (45)
Nurses	1717 (33)	427 (30)	2144 (33)
Other Health Workers *	1020 (20)	470 (33)	1490 (23)
**Age at vaccination—y**			
Median	39	–	39
Range	20–76	–	20–76

y, years. * Other Health Workers category includes midwiferies, healthcare assistants, pharmacists, psychologists, laboratory technicians, radiology technicians, physiotherapists, logopedists, perfusionists, neurophysiopathology technicians, biologists, environment and workplace prevention technicians, dieticians, orthoptist, audiometrists, occupational therapists, and neurodevelopmental disorders therapists.

**Table 2:** Detailed FPG safety results compared to the SPC of Comirnaty.

System Organ Class	Very Common (≥1/10)	Common (≥1/100 to <1/10)	Uncommon (≥1/1000 to <1/100)	Rare (≥1/10,000 to <1/1000)	Not Known ^§^
Blood and lymphatic system disorders	–	–	Lymphadenopathy **15%**	–	–
Immune system disorders	–	–	Hypersensitivity reactions (e.g., rash, pruritus, urticaria, angioedema) **10%**	–	Anaphylaxis NR
Metabolism and nutrition disorders	–	–	Decreased appetite **2%**	–	–
Psychiatric disorders	–	–	Insomnia **2%**	–	–
Nervous system disorders	Headache 39% (ref. >50%) **Paresthesia** **24%**	**Vertigo** **5%**	Lethargy NR	Acute peripheral facial paralysis NR	–
Cardiac disorders	Tachycardia **8%** (ref. >60%)	–	–	–	Myocarditis; Pericarditis 0.66%
Respiratory disorders	**Dyspnea** **4%**		–	–	–
Gastrointestinal disorders	Diarrhea 5%	Nausea **13%** Vomiting 3% **Abdominal pain** **3%**	–	–	–
Skin and subcutaneous tissue disorder			Hyperhidrosis **1%** Night sweats **1%**		
Musculoskeletal and connective tissue disorders	Arthralgia 28% (ref. >20%) Myalgia 28% (ref. >40%)	–	Pain in extremity 1%	–	–
General disorders and administration site conditions	Injection site pain 26% (ref. >80%) Fatigue 3% (ref. >60%) Chills 37% (ref. >30%) Pyrexia 39% (ref. >10%) Injection site swelling 1% (ref. >10%)	Injection site redness 3%	Malaise 6% Injection site pruritus 3% Asthenia 30%	–	Extensive swelling of vaccinated limb 0.66% Facial swelling 5%

The percentages that do not align to the category were highlighted in bold as well the unexpected symptoms detected. ^§^ cannot be estimated from the available data. – indicates that no symptoms were expected for those categories.

## Data Availability

The data that support the findings of this study are available on request from the corresponding author, M.C.N.
